# Therapeutic Perspectives for the Perioperative Period in Oral Squamous Cell Carcinoma (OSCC)

**DOI:** 10.3389/froh.2021.764386

**Published:** 2022-01-11

**Authors:** Antoine Galmiche, Zuzana Saidak, Jérémie Bettoni, Martial Ouendo, Sylvie Testelin

**Affiliations:** ^1^EA7516 CHIMERE, Université de Picardie Jules Verne, Amiens, France; ^2^Service de Biochimie, Centre de Biologie Humaine, Centre Hospitalier Universitaire (CHU) Amiens, Amiens, France; ^3^Service de Chirurgie Maxillo-Faciale, Centre Hospitalier Universitaire (CHU) Amiens, Amiens, France; ^4^Service d'Anesthésie Réanimation, Centre Hospitalier Universitaire (CHU) Amiens, Amiens, France

**Keywords:** perioperative period, surgical stress, OSCC (Oral Squamous Cell Carcinoma), inflammation, coagulation, anesthesia

## Abstract

The perioperative period is the relatively short window of time, usually measured in days or weeks, around the surgical procedure. Despite its short duration, this time period is of great importance for cancer patients. From a biological point of view, the perioperative period is complex. Synchronous with primary tumor removal, surgery has local and distant consequences, including systemic and local inflammation, coagulation and sympathetic activation. Furthermore, the patients often present comorbidities and receive several medical prescriptions (hypnotics, pain killers, anti-emetics, hemostatics, inotropes, antibiotics). Because of the complex nature of the perioperative period, it is often difficult to predict the oncological outcome of tumor resection. Here, we review the biological consequences of surgery of Oral Squamous Cell Carcinoma (OSCC), the most frequent form of primary head and neck tumors. We briefly address the specificities and the challenges of the surgical care of these tumors and highlight the biological and clinical studies that offer insight into the perioperative period. The recent trials examining neoadjuvant immunotherapy for OSCC illustrate the therapeutic opportunities offered by the perioperative period.

## The Perioperative Period and Its Oncogenic Relevance

**Figure 1:**
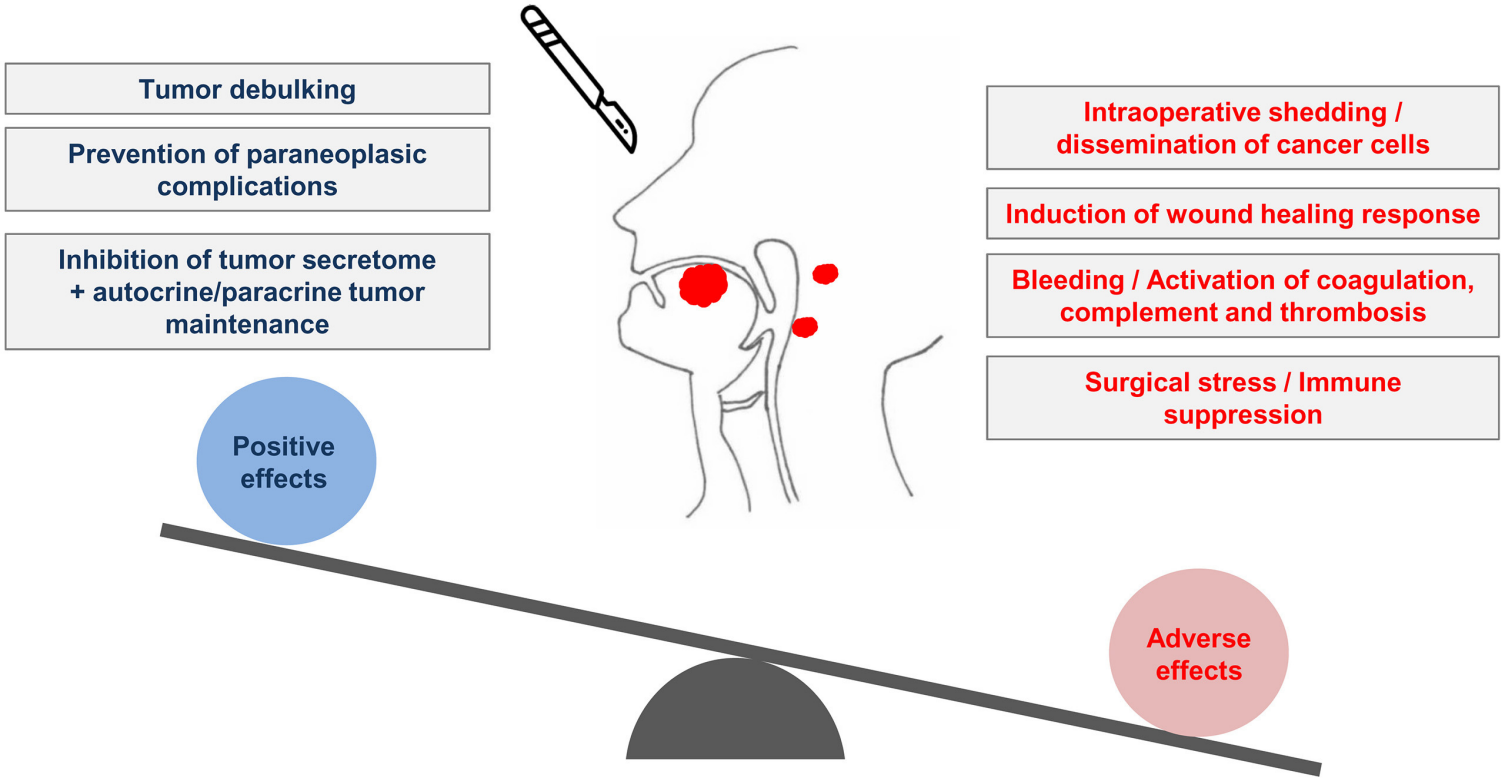
The perioperative period and the conflicting biological influences of surgery on tumors of the oral cavity. Surgery of OSCC produces both positive and adverse effects. The removal of the tumor leads to the beneficial effects of debulking and inhibition of the tumor secretome. Conversely, OSCC surgery can lead to neoplastic cell dissemination. The concomitant surgical stress and the activation of coagulation as a consequence of bleeding may create biological conditions prone to post-surgical recurrence of OSCC.

Surgical tumor removal is an essential procedure in cancer management, and for most patients it is the only curative procedure available. The perioperative period is the time window, usually measured in days or weeks, around the surgical procedure. Despite its relatively short duration, the perioperative period is very important for the outcome of cancer [1, 2]. The aim of surgery is usually to achieve complete tumor removal with R0 clear surgical margins. Even in this situation however, the surgical procedure has multiple opposing influences on the oncological outcome. During surgery, cancer cells can be mobilized or left behind, potentially paving the way to tumor recurrence. Surgical resection however drastically reduces tumor burden and prevents autocrine/paracrine tumor signaling. One positive effect of tumor debulking may come from reduced secretion of growth factors, cytokines and hormones by cancer cells, since these cells usually have an altered secretome with potential systemic consequences [3]. The beneficial effects of tumor debulking may however be negated by, among other events, the proinflammatory and neuroendocrine consequences of the surgical procedure, as discussed below (Figure 1). Due to the complex consequences of these simultaneous events, it is always difficult to reliably predict the oncological outcome of surgery. This uncertainty has led some authors to compare the perioperative period to a Russian roulette [4]. Here, we discuss the challenges of the study of the perioperative period and the opportunities for therapeutic improvement against Oral Squamous Cell Carcinoma (OSCC) (Table 1).

**Table 1:** An overview of the main perioperative biological events, the ligands and receptors involved and the potential therapeutic interventions.

**Periooperative event**	**Ligand/receptor involved**	**Possible therapeutic intervention**
Inflammation/wound healing	DAMPs, PAMPs/TLRs	NSAIDs
	Cytokines/Cytokine Receptors	Control of oral microbiote
		Minimally-invasive surgery
Bleeding, coagulation	Coagulation proteases/PAR1-4	Personalized anticoagulation
Stress, pain	cortisol/Glucocorticoid receptor	Stress coping procedures
	Catecholamines/β2AR	Perioperative β-blockers
	Opioids/opioid Receptors	Effective pain control, optimized antalgic regimen
Immune suppression	corticosteroids, malnourishment	ERAS protocols
		Neoadjuvant ICI

## The Difficulties of Studying the Perioperative Period in Oral Cavity Tumors

While numerous studies have been devoted to the medical management of advanced stages of cancer, few studies have examined the oncological relevance of the perioperative period. An even more restricted number of studies have addressed the biological impact of the perioperative period on tumors of the oral cavity, including OSCC. The task is complex, due to the large number of parameters that influence the outcome of OSCC resection: cancer patients are often old, frail, undernourished and with comorbidities, such as anemia or diabetes. They often receive multiple drugs with different modes of action (hypnotics, pain killers, anti-emetics, hemostatics, inotropes, antibiotics, etc.) [5]. The intricate nature of the reconstructive surgical procedures is also an element of complexity. It is recognized that the extent of surgical resection for OSCC is considered in view of the need to preserve phonation, deglutition and the overall quality of life. Progress in surgical reconstruction, anesthetic and surgical techniques, including microvascular reconstruction, currently allow more radical surgical procedures for advanced OSCC. The use of free flap reconstruction illustrates the progress in the field, but it comes with its own perioperative follow-up and complications [6]. Lastly, precisely defining which perioperative events are important in OSCC patients requires properly-powered prospective studies with a long follow-up in this patient population treated with curative intent. For this reason, most evidence available to date is based on pre-clinical findings and retrospective examination of clinical data.

## Key Perioperative Events and the Relevant Biological Parameters

### Inflammation and Wound Healing

As a consequence of the unavoidable destruction of cells and tissues, surgical procedures systematically induce an inflammatory response [7]. Tissue damage can be induced by mechanical force applied to the tissues. It can also be of circulatory origin (due to ischemia and reperfusion). From a fundamental perspective, the inflammatory response prepares the operated tissues for subsequent wound healing [8]. Cell destruction leads to the systemic release of Damage-Associated Molecular Patterns (DAMPs), also known as alarmins, i.e., cellular molecules that transduce a danger signal by activating conserved receptors of the innate immune system, such as the Toll-Like Receptors (TLR) [7]. DAMPs owe their acronym to their effects that are reminiscent of those released by Pathogen (Pathogen-Associated Molecular Patterns, PAMPs). Depending on the type of tissue and the extent of cellular destruction (apoptosis, necrosis or cellular stress), a repertoire of DAMPs is released and then bind to and activate TLR on the surface of the different cell types that constitute the tumor microenvironment (TME) of OSCC. In the oral cavity, the presence of an oral microbiote is an important parameter that accounts for the presence of PAMPs that may further stimulate local inflammation [9]. The effects of surgery may interact and possibly cumulate with preoperative inflammation, whose negative impact in OSCC is reflected by routine laboratory analyses, such as serum concentrations of C-reactive protein (CRP) [10]. Inflammation plays a complex role in tumor initiation, progression and response to treatment [11]. Importantly, several studies provide a rationale for the stimulation of OSCC growth by inflammation, both through direct effects on cancer cells [12] and indirect (TME-mediated) mechanisms [13]. Besides fueling tumor growth, inflammation stimulates tumor neoangiogenesis, prepares the pre-metastatic niche and exerts specific effects on remote tissues, paving the way to post-surgical recurrence of OSCC [14]. A better description of the complex cellular interactions established within the TME of OSCC is required to better understand how the systemic inflammation induced by surgery intersects with tumor progression [15].

Non-Steroidal Anti-Inflammatory Drugs (NSAIDs) and aspirin directly target OSCC cells and reduce their viability and their production of inflammatory mediators [16, 17]. Counteracting inflammation with NSAIDs may limit the dissemination of cancer cells during the perioperative period. However, inappropriate use of NSAIDs may delay healing, favoring anastomotic leaks and leading to poor tissue repair [18]. Unfortunately, there is no available evidence based on randomized clinical trials to guide the use of NSAIDs in OSCC treated by primary surgery. Importantly, a subpopulation of HNSCC with *PIK3CA* gene alterations may get a better clinical benefit from NSAIDs [19]. This is potentially important, as this observation paves the way to personalized use of NSAIDs during the perioperative period [19, 20]. Finally, the existence of a correlation between tissue damage and inflammation provides a rationale for the use of minimally-invasive procedures whenever they can be safely applied, as was for example shown with more selective neck dissection (SND). Along this line, Fan *et al*. recently examined the serum inflammatory profile of cN0 OSCC patients treated with an open surgical technique *vs*. endoscopically-assisted neck dissection [21]. This interesting, yet preliminary study, suggests that minimally-invasive procedures may indeed decrease the perioperative peak of IL-6 in the serum. More studies are required to validate these conclusions in different configurations, and to address the potential oncological benefits of preventing perioperative inflammation.

### Bleeding and Coagulation

Surgical procedures lead to blood loss and activate coagulation. The risk and amount of bleeding depend on the type and complexity of surgical procedures, as well as on many other parameters, such as hemodilution, platelet dysfunction, and the use of anticoagulants and platelet inhibitors [22]. OSCC patients often present anemia, possibly related to malnutrition and chronic inflammation. The frequent occurrence of liver cirrhosis, due to the frequent chronic alcohol consumption in this population, can potentially compromise the capacity of the liver to produce both pro- and anti-coagulant proteins and predisposes to hemorrhagic accidents as well as to thrombotic events [23].

Importantly, solid tumors almost systematically trigger a hypercoagulant state accounted for by altered expression of key actors of coagulation and fibrinolysis [24]. The term “coagulome” highlights the interest of systems biology for the study of these hemostatic complications [25, 26]. The coagulome of OSCC is characterized by the coexistence of high expression of the Tissue Factor, the main activator of coagulation and urokinase-type Activator of Plasminogen (uPA), the main activator of fibrinolysis [24]. When activated during bleeding, the coagulation cascade activates the Protease-Activated Receptors-1 to−4 (PAR1-4). The PARs are a family of G-Protein coupled Receptors that are often described as coagulation sensors, since their mode of action includes a proteolysis step induced by thrombin or other coagulation proteases. The expression of PAR2 was recently reported to transduce mitogenic and proinvasive signals in OSCC cells [27, 28]. The activation of PAR2 may directly boost tumor growth after surgery. Coagulation is well known to exert proinflammatory effects, for example by activating the complement and the production of the anaphylatoxins C3a and C5b. The expression of other PARs on cells of the TME may also produce subtle immune modulatory effects, as was recently suggested in other tumor types [29]. A polymerized fibrin matrix deposited during healing after surgery, as a consequence of coagulation, may serve as an adherence site for cancer cells [30]. It may also protect circulating tumor cells from immune cells in the circulation. Circulating tumor cells (CTC) are detected in 12.5% of operated OSCC patients [31]. Detection of CTC represents an independent risk factor of recurrence and clotting may be a protective mechanism for these cells, ensuring their perioperative dissemination. Recent studies showing the prognostic value of biomarkers of hemostasis in surgical OSCC [32, 33] open the interesting possibility of better and more personalized anticoagulation strategies during the perioperative period.

### Surgical Stress, Catecholamines, and Cortisol

Surgical stress is a consequence of multiple physical and psychological events that occur around the time of surgery. The hypothalamus-pituitary-adrenal (HPA) axis and the sympathetic nervous system are activated, leading to the release of cortisol and catecholamines, respectively. Catecholamines activate α and β-adrenoreceptors (AR), a family of G-protein coupled receptors broadly expressed in normal and pathological cells and tissues. The β2AR is predominantly expressed by OSCC [34], but variable and even conflicting observations have been made regarding the effects of catecholamines on OSCC cells. β2AR antagonists were reported to block the growth of OSCC cells *in vitro* and *in vivo* in immune-deficient mouse models, suggesting a potential direct stimulation of cancer cell growth by catecholamines [35]. A recent study also found that β2AR activation can prevent the acquisition of a mesenchymal and motile phenotype by OSCC cells [36]. The direct consequences of β2AR receptor activation in OSCC cells may vary depending on the tumor genomic background, but the effects of catecholamines may be more consistent in cells of the TME. In a model of breast carcinogenesis, sustained exposure of tumor tissue to catecholamines was found to stimulate lymphatic drainage of tumors and cancer cell dissemination [37]. The activation of β2AR receptors might also occur in remote tissues and contribute to the preparation of a permissive metastatic niche, as suggested for example in studies examining the impact of catecholamines on bone tissue [38]. Recent clinical trials in breast and colorectal tumors provide a proof of principle that the perioperative use of β-blockers (propranolol) is safe, in addition to showing that it might have a favorable effect on tumor biomarkers reflecting tumor metastatic potential [39, 40].

Importantly, the HPA axis is an integral part of immune regulation. Corticosteroids are also widely used as anti-inflammatory drugs in cancer patients. The concept of neuroimmunomodulation puts emphasis on the strong, mutual connections that exist between neural and immune cell functions [41]. Although still a research field, glucocorticoids do not simply inhibit all cell-mediated immune responses. Besides their well-recognized inhibitory action on macrophages, T cells and NK cells, their circadian secretion controls diurnal oscillations of the distribution and response of T cells [42]. Although no studies to date have examined the impact of the timing of surgical procedures in OSCC patients, it might be an important determinant of postoperative recovery. A recent study shows an intrinsic morning-afternoon variation in hypoxia-reoxygenation tolerance, concomitant with circadian gene expression in cardiovascular tissues [43]. A recent experimental study in a model of bone surgery also suggests the potential benefit of the application of chronobiology in enhancing post-operative recovery [44]. An overview of chronobiology of OSCC is beyond the scope of the present review, but both the tumor and the patient might differentially respond to morning or evening surgery.

### Anesthesics and Analgesics

Surgical cancer patients receive general, sometimes local or regional anesthesia, and analgesia [45]. General anesthesia typically relies on total intravenous anesthesia (propofol) or volatile inhaled anesthesia (isoflurane). The problems raised by the clinical tolerance to general anesthesia in OSCC patients can be found elsewhere [46, 47]. Besides the different immediate post-operative characteristics induced by the choice of anesthesia [48], specific protocols may change the risk of post-surgical recurrence of cancer, as suggested in various preclinical models (review in Cata et al. [45]). The different anesthetics and/or analgesic regimen used in oral surgery can produce direct effects on OSCC cells [49–51]. They can also act indirectly on immune cells and the TME. In the absence of solid data examining this possibility in OSCC, we refer the reader to studies and consensus established for other tumors regarding the possible effects (positive or negative) of anesthesics/analgesics on post-surgical recurrence of cancer [52–54].

Arguably, opioids provide a good illustration of an analgesic intervention that could affect the risk of post-surgical recurrence in OSCC. Fentanyl and its derivatives are an essential component of perioperative analgesia for OSCC patients. Patients receiving curative intent surgery for OSCC are also at risk of persistent use of opioids [55]. Their potent analgesic efficacy is produced through the stimulation of the μ opioid receptor (MOR), a receptor that is expressed by a subset of OSCC cells [56]. Opioid agonists promote the invasive growth of MOR-positive OSCC *in vitro* and stimulate tumor formation in mice [56]. Because only few clinical studies have examined the impact of opioids on post-surgical recurrence of OSCC, it remains difficult to draw definitive conclusions and to issue solid clinical recommendations. After adjusting for clinical covariates, Patino et al. observed a trend between the quantities of opioids administered during surgery and recurrence free-survival/overall survival in operated OSCC patients that however did not reach statistical significance [57].

## Studying the Perioperative Window for the Development of Better Therapeutics Against OSCC

A substantial number of practical improvements were made over the past years regarding surgical care. Besides the progress of minimally-invasive surgery, robotics and ≪ augmented ≫ surgery [58], the implementation of Enhanced Recovery After Surgery (ERAS) protocols [59–61] represents a significant attempt to modernize surgical procedures. The ERAS protocols constitute a collection of supportive measures that aim to reduce some of the complications and negative effects of surgery, such as surgical stress. Importantly, ERAS protocols aim to reduce the early complications of surgery and facilitate the patient's discharge. There is currently no evidence that the application of ERAS protocols can favorably impact the oncological prognosis of OSCC, despite their positive effects on a number of blood markers (CRP, albumin and neutrophil-to-lymphocyte ratio) [60]. This will be an important aim for future studies.

Prehabilitation is an important concept, highlighting the necessity of preparing patients upstream of surgery. A strong rationale for its relevance comes for the significant impact of pre-existing morbidities on tumor mortality, especially diabetes [47, 62] and malnourishment [63, 64]. Nutritional support was shown to extend the overall survival and recurrence free-survival of malnourished patients with HNSCC [65]. Nutritional support with essential nutrients and amino-acids, such as L-Arginine, improves wound healing and prevents infection after surgery [66, 67], but it is not known whether they have an impact on the oncological outcome of surgery. How comorbidities shape tumor biology and modify the TME is an emerging field of research that promises to give new insight on post-surgical recurrence [68].

The possibility of targeting OSCC with neoadjuvant treatment has long been a topic of debate. The failure of neoadjuvant chemotherapy to increase the survival of locally-advanced resectable OSCC vs. up-front surgery demonstrates the complex nature of the problem [69]. Importantly however, the recent trials examining neoadjuvant treatment with immune checkpoint blockers (ICB) bring new elements to this discussion [70, 71]. The antibodies pembrolizumab and nivolumab, that neutralize the interaction between the immune regulatory molecule PD1 (Programmed death-ligand 1) and its ligand PD-L1, were the first clinically-approved ICB for recurrent/metastatic HNSCC [72]. Two recent phase 2 trials examined their use, either alone or in combination with other ICB, in the neoadjuvant context in OSCC [73, 74]. In both trials, neoadjuvant immunotherapy administered as one or two cycles 3 weeks prior to surgery led to downstaging in more than half of OSCC [73]. Interestingly, a number of preclinical studies suggest that: (i) the presence of the tumor may provide antigenic stimulation contributing to better efficacy of ICB prior to surgery; (ii) ICB may prevent cancer cell dissemination induced by the surgical procedure [70, 71]. Evidence is still lacking for the oncological value of neoadjuvant ICB, but the prospect of harnessing cancer immunotherapy during the perioperative period is exciting [75]. These studies do not only challenge the current surgical practice of proposing rapid upfront resection of OSCC, they will also likely lead to novel discoveries regarding the biology of OSCC in the perioperative period.

## Data Availability Statement

The original contributions presented in the study are included in the article/supplementary material, further inquiries can be directed to the corresponding author/s.

## Author Contributions

AG and ZS wrote the initial version of the manuscript. JB, MO, and ST critically read and edited the manuscript. All authors contributed to the article and approved the submitted version.

## Funding

This work was suppoeted by Ligue contre le Cancer, comité de la Somme, and CHU Amiens Picardie.

## Conflict of Interest

The authors declare that the research was conducted in the absence of any commercial or financial relationships that could be construed as a potential conflict of interest.

## Publisher's Note

All claims expressed in this article are solely those of the authors and do not necessarily represent those of their affiliated organizations, or those of the publisher, the editors and the reviewers. Any product that may be evaluated in this article, or claim that may be made by its manufacturer, is not guaranteed or endorsed by the publisher.
